# Correlation between A3243G and G9053A mtDNA mutations and ATP levels in diabetes mellitus patients using qPCR and electrochemical aptasensors

**DOI:** 10.5599/admet.2767

**Published:** 2025-06-12

**Authors:** Iman Permana Maksum, Rahmaniar Mulyani, Yeni Wahyuni Hartati, Irkham, Fanny Rizki Rahmadanthi, Serly Zuliska, Toto Subroto

**Affiliations:** 1 Department of Chemistry, Faculty of Mathematics and Natural Sciences, Universitas Padjadjaran, Sumedang, 45363, Indonesia; 2 Department of Chemistry, Faculty of Sciences and Informatics, Universitas Jendral Achmad Yani, Cimahi, 40525, Indonesia

**Keywords:** Electrochemical biosensor, mitochondrial diabetes diagnostics, mitochondrial mutations, nucleic acid amplification

## Abstract

**Background and purpose:**

Mitochondrial DNA (mtDNA) mutations can impair oxidative phosphorylation and ATP production, potentially contributing to the pathogenesis of type 2 diabetes mellitus (T2DM). This study aimed to investigate the relationship between mtDNA mutations and ATP levels in blood and urine samples from T2DM patients.

**Experimental approach:**

Samples from 60 patients (30 with T2DM + mitochondrial disease [MD] phenotype and 30 with T2DM alone) were analysed. mtDNA mutations A3243G and G9053A were detected using qPCR with dual-labeled probes (FAM for mutant, HEX for wild type) based on *C*q comparisons. ATP concentrations were measured using a screen-printed carbon electrode (SPCE)-based electrochemical aptasensor.

**Key results:**

The A3243G mutation was more frequent and had higher heteroplasmy levels than G9053A, particularly in the T2DM + MD group. Although no statistically significant differences in ATP levels were observed between groups, descriptive ranges showed lower ATP concentrations in the T2DM + MD group (314 to 919 μM) compared to the T2DM group (746 to 1130 μM), both below the physiological range (1.500 to 1.900 μM). A similar pattern was found for A3243G mutation levels, while G9053A levels overlapped between groups. Two-way ANOVA showed a significant association between mutation presence and reduced ATP levels.

**Conclusion:**

The A3243G mutation may be more directly associated with mitochondrial ATP depletion in T2DM, while the role of G9053A remains inconclusive. This study highlights the potential of combining molecular and electrochemical tools to assess mitochondrial contributions in diabetes.

## Introduction

The mitochondrial genome is a circular DNA molecule that encodes 13 protein-coding genes, 2 ribosomal RNAs (rRNAs), and 22 transfer RNAs (tRNAs). Due to the absence of histone protein protection and limited DNA repair mechanisms, mitochondrial DNA (mtDNA) is highly susceptible to mutations [1-3]. These mutations, particularly in the polymorphic regions of the mtDNA respiratory complex, significantly impact ATP synthesis and cellular energy production [4]. For example, lymphocytes from patients with maternally inherited hypertension exhibited the C4467A mutation (MT-TM gene), which resulted in a 114.5 % increase in reactive oxygen species (ROS) production and a 26.4 % reduction in ATP levels compared to control cells [5].

Mutations in mitochondrial DNA are linked to a variety of disorders, including mitochondrial diseases (MD) [6]. MD is characterized by phenotypic traits such as a maternal inheritance pattern, which reflects the transmission of mtDNA mutations through the maternal lineage [7,8]. Additionally, neuromuscular symptoms are commonly observed, stemming from the high energy demands of muscle tissues, which depend on mitochondrial function for efficient ATP production [9,10].

Among the mtDNA mutations, the A3243G mutation in the tRNA^Leu(UUR)^ gene is one of the most extensively studied, particularly for its role in mitochondrial diseases such as MELAS, MID, and maternally inherited diabetes and deafness (MIDD) [11,12], with a prevalence of 0.95 to 16.3 per 100,000 individuals [3,13]. It impairs tRNA function, disrupting protein assembly and reducing ATP production. Structurally, the mutation destabilizes the tRNA arm by disrupting the A14-U8 bond and introduces a palindromic hexanucleotide sequence (5-GGGCCC-3), promoting dimer formation [14]. These changes impair aminoacylation, codon recognition, and post-transcriptional methylation, leading to mitochondrial dysfunction and insufficient ATP synthesis, which inhibits K^+^ channel opening and insulin secretion in response to hyperglycemia, contributing to T2DM [14-17].

A novel G9053A mutation was identified in four T2DM patients with cataracts, all carrying the A3243G mutations [10], and has been reported in silico to disrupt the proton channel of ATP synthase through the S167N substitution in the ATPase6 gene, impairing ATP production and contributing to T2DM and cataract pathophysiology [18]. Despite these individual findings, no prior study has investigated the combined effect of A3243G and G9053A mutations on ATP levels in T2DM.

Various techniques, including qPCR, PCR-RFLP, and allele-specific PCR, have been developed to detect mtDNA mutations [19-22]. qPCR is particularly favoured for its rapid, sensitive, and accurate detection, effectively quantifying low-level heteroplasmy in A3243G mutations. Recent advancements in qPCR assays have enabled the detection of A3243G mutations with sensitivities as low as 0.1 %, providing a reliable approach for analysing mitochondrial dysfunction linked to T2DM [21].

Aptamers, short oligonucleotides with high target specificity, have garnered interest in biosensor applications, including ATP detection [23,24]. Previous research has shown that SPCE-based aptasensors are effective for ATP quantification [25], demonstrating both high specificity and stability. In silico analyses of aptamer-ATP interactions further confirmed strong hydrogen bonding, electrostatic interactions, and maintained aptamer structural integrity, contributing to reliable and precise ATP measurement [26].

This study investigates the relationship between A3243G and G9053A mtDNA mutations and ATP levels in individuals with T2DM, including those with and without mitochondrial dysfunction (MD), using qPCR for mutation detection and an optimized SPCE-based aptasensor for ATP measurement. Samples were selected based on clinical inclusion criteria, including maternal inheritance, neuromuscular symptoms, BMI ≤25 kg/m^2^, and absence of ketoacidosis. These criteria were chosen to ensure the participants had relevant disease characteristics, minimizing confounding factors and focusing on mitochondrial dysfunction in diabetes. The findings provide insight into mitochondrial dysfunction in diabetes and explore the use of integrated molecular, and biosensor approaches to improve diagnostic strategies for mitochondrial diabetes.

## Experimental

### Material

Materials used in this study included 100 bp DNA ladder (Promega), acetic acid (Sigma Aldrich), agarose (Sigma Aldrich), ApaI and HhaI restriction enzymes (New England Biolabs), EDTA (Sigma Aldrich), ethanol (Merck), forward and reverse primers (Macrogen), GelRed (Promega), K_3_[Fe(CN)_6_] (Sigma), KCl (Merck), loading dye (Promega), mutant probe (Macrogen), normal probe (Macrogen), nuclease-free water (Promega), PCR mix (Tiangen Biotech), Proteinase K (Promega), qPCR mix (THUNDERBIRD™ Probe qPCR Master Mix), SDM forward and reverse primers (Macrogen), the aptasensor, Tris-HCl (Sigma Aldrich), and Vazyme DNA/RNA extraction kit 96 (Vazyme). Additional materials included TE buffer, physiological NaOH, and lysis buffer.

### Procedure

#### Patient samples

Blood and urine samples were collected from 60 patients diagnosed with T2DM, with and without mitochondrial disease (MD), based on medical records at RS. Dustira Cimahi. This study was approved by the Universitas Padjadjaran Research Ethics Committee (Approval number: 533/UN6.KEP/EC/2022) and conducted in accordance with the Declaration of Helsinki. All patients provided written informed consent and were randomly selected from outpatients at RS. Dustira Cimahi. Participants with the T2DM + MD phenotype were characterized by maternal inheritance (a direct maternal hereditary factor), neuromuscular symptoms, BMI ≤25 kg/m^2^, and non-ketoacidosis, while T2DM participants exhibited the opposite characteristics.

#### Isolation and purification of mitochondrial DNA

Mitochondrial DNA (mtDNA) was extracted from peripheral blood and urine to obtain DNA of sufficient purity and quality for qPCR analysis. Lymphocytes were isolated from 200 μL of whole blood by repeated washing with 1000 μL TE buffer (10 mM Tris-HCl, pH 8.0; 0.5 mM EDTA), followed by centrifugation at 12,000 rpm for 1 minute at 0 °C, until a white pellet was visible. Epithelial cells were collected from 5 mL of fresh urine by centrifugation at 14,000× *g* for 10 minutes at 4 °C, washed with 0.85% physiological NaOH, and subjected to multiple washes with TE buffer (up to five times). Each pellet was then resuspended in a lysis buffer composed of 50 mM Tris-HCl (pH 8.5), 1 mM EDTA, 0.04 mg/mL proteinase K, and 0.5 % Tween-20, incubated at 56 °C for 1 hour and at 95 °C for 5 minutes. The lysates were centrifuged at 20,000× *g* for 5 minutes, and the resulting supernatants were used as templates for qPCR using mtDNA-specific primers. Samples were divided into two groups: T2DM with MD phenotype (n = 30) and T2DM without MD phenotype (n = 30).

#### Identification of A3243G and G9053A mutations

Primers were designed using the Homo sapiens mitochondrial tRNALeu gene (GeneID: 4567) sequence obtained from the NCBI website (*http://www.ncbi.nlm.nih.gov*) and Perlprimer software (Table 1). Probes were designed as described previously [21] (Table 1), with each probe labelled with a fluorescent compound at the 5' end as a reporter and a Black Hole Quencher (BHQ) at the 3' end as a quencher. HEX dye was used for the normal probe, and FAM for the mutant probe. The oligonucleotide sequence of the probe was differentiated by one base at the mutation point to be analysed, with the modified base located in the middle of the probe sequence to increase probe hybridization specificity to the DNA template.

**Table 1.**
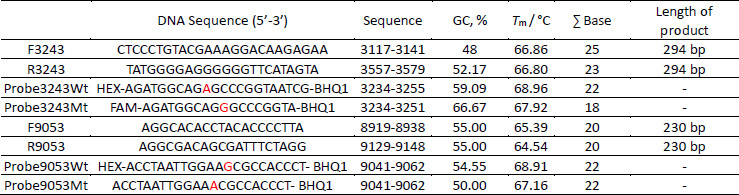
Nucleotide sequences of the primers and probes for qPCR

F: forward primer; R: reverse primer; GC: guanine-cytosine content of the primer or probe sequence; ∑ Base: total number of nucleotides in each primer or probe; *T*_m_: melting temperature

The qPCR conditions and DNA template concentration were optimized to ensure sufficient sensitivity and specificity for identifying the A3243G and G9053A mutations [27]. The reaction mixture consisted of 10 μL of THUNDERBIRD™ Probe qPCR Master Mix, 0.2 μL of forward primer, 0.2 μL of reverse primer, 0.5 μL each of the normal and mutant probes at a concentration of 0.2 and 2 μL of DNA template. The total volume was adjusted to 20 μL. The qPCR reaction involved creating stocks of Probe3243Mt, Probe323Wt, Probe9053Mt, and Probe9053Wt, using isolated DNA as a template at a concentration of 250 ng/μL.

After determining the optimal DNA template concentration, amplification was performed using PCR conditions based on previous research [22]: activation at 94 °C for 5 minutes, followed by 30 cycles of 94 °C for 30 seconds (denaturation), 56 °C for 30 seconds (annealing), and 72 °C for 50 seconds (elongation), with a final elongation step at 72 °C for 10 minutes. The cycling conditions for identifying the G9053A mutation were based on a previous study [20]: activation at 95 °C for 5 minutes, followed by 30 cycles of 95 °C for 30 seconds (denaturation), 55 °C for 30 seconds (annealing), and 60 °C for 30 seconds (elongation), with a final elongation step at 60 °C for 10 minutes.

### ATP quantification using an aptasensor

Blood (200 μL) and urine (5 mL) samples were processed immediately after collection. Urine samples were centrifuged at 14,000× *g* for 10 minutes, and the resulting pellet was washed sequentially with 1,000 μL of 0.85 % NaOH, followed by 1,000 μL of TE buffer (10 mM Tris-HCl, 0.5 mM EDTA, pH 8.0). Blood samples were washed with 1,000 μL of TE buffer and centrifuged until a white pellet was obtained. Both blood and urine pellets were resuspended in nuclease-free water and subjected to lysis with a buffer containing 50 mM Tris-HCl (pH 8.5), 1 mM EDTA, 0.04 mg/mL proteinase K, and 0.5 % Tween-20. Lysis was performed at 56 °C for 1 hour, followed by enzyme inactivation at 95 °C for 5 minutes. After lysis, the samples were centrifuged at 20,000× *g*, and the supernatants were collected for ATP quantification. ATP levels were determined using differential pulse voltammetry (DPV) with an electrochemical aptasensor. The analysis was carried out in 10 mM K_3_[Fe(CN)_6_] with 0.1 M KCl, using a potential range of -1.0 to +0.7 V, a scan rate of 0.008 V/s, *E*_step_ of 0.004 V, *E*_pulse_ of 0.025 V and *t*_pulse_ of 0.05 s. The resulting peak currents were used to quantify ATP levels, with the measurements performed as described previously [25].

### Statistical analysis of the correlation between the mutations and levels

The participants were divided into two groups: T2DM with a diabetes mellitus (DM) phenotype and T2DM without a DM phenotype. The groups were further categorized based on the type of sample: blood or urine. Shapiro-Wilk and Levene's tests were performed to assess the correlation between the phenotype and ATP levels, with the significance level set at *α* = 5 %.

## Results and Discussion

### Identification of A3243G and G9053A mutations by qPCR

The detection of A3243G and G9053A mutations was carried out using specific primer–probe sets designed to distinguish mutant from wild-type mitochondrial DNA. As listed in Table 1, the A3243G mutation was analysed using primers F3243 and R3243, with Probe3243Wt (HEX-labelled) targeting the wild-type allele and Probe3243Mt (FAM-labelled) detecting the mutant variant. Similarly, detection of the G9053A mutation employed primers F9053 and R9053, with Probe9053Wt and Probe9053Mt labelled with HEX and FAM, respectively. Amplification curves and quantification cycle (*C*_q_) values were used to interpret the fluorescence signals, where lower *C*_q_ values indicated higher concentrations of the respective DNA target. As shown in Figures S1 and S2, fluorescence from HEX signified the presence of wild-type DNA, while FAM fluorescence reflected the abundance of mutant sequences. The consistent separation of *C*_q_ values between HEX and FAM signals across samples confirmed the specificity of each probe set in distinguishing between normal and mutant alleles for both mutations.

The qPCR analysis showed that 12 samples from the T2DM + MD phenotype group had lower *C*_q_ values for FAM compared to HEX for both the A3243G and G9053A mutations, indicating the presence of these mutations. As shown in Table 2, values written in red font represent C_q_ values for FAM that are lower than the corresponding HEX values, highlighting samples in which the mutation was detected. Specifically, seven blood samples and two urine samples exhibited lower FAM values for the A3243G mutation, while eight blood samples and seven urine samples showed lower FAM values for the G9053A mutation. Furthermore, three samples were identified as carrying both the A3243G and G9053A mutations.

**Table 2.**
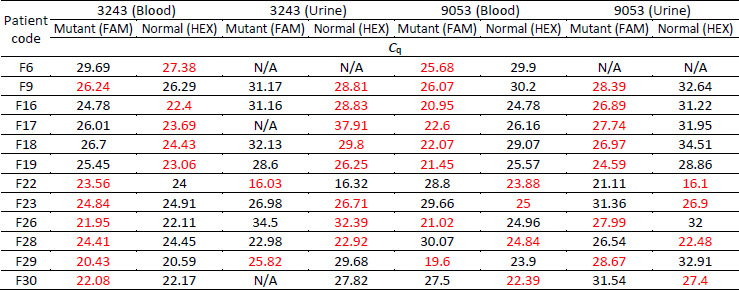
*C*_q_ values of patients in the T2DM + MD phenotype group

Further analysis of the amplification curves from the T2DM group identified six samples with lower *C*q values for FAM than HEX, indicating the presence of A3243G or G9053A mutations (Table 3). One urine sample showed a lower FAM signal for A3243G, while no corresponding mutations were detected in the blood. In contrast, five samples, both blood and urine, exhibited lower FAM signals for G9053A.

**Table 3.** *C*_q_ value from qPCR amplification results in patients in the T2DM group

Patient code	3243 (Blood)		3243 (Urine)		9053 (Blood)		9053 (Urine)	
	Mutant (FAM)	Normal (HEX)	Mutant (FAM)	Normal (HEX)	Mutant (FAM)	Normal (HEX)	Mutant (FAM)	Normal (HEX)
*C*_q_								
NF7	27.52	26.11	24.9	28.12	31.47	26.3	33.44	28.77
NF13	30.1	27.93	29.22	26.81	26.3	30.64	25.93	30.2
NF15	26.37	24.33	32.6	30.18	22.14	27.58	27.29	34.6
NF17	25.43	23.13	27.57	25.15	21.56	25.99	23.12	27.45
NF26	25.42	23.06	29.1	26.68	20.39	27.59	23.89	31.37
NF30	29.45	27.1	31.38	29.03	25.37	29.93	25.48	29.7

None of the T2DM samples carried both mutations. The T2DM + MD phenotype group showed a higher frequency of mtDNA mutations, with 12 patients carrying either A3243G or G9053A, compared to 6 patients in the T2DM group. This trend suggests a stronger association between mtDNA mutations and the MD phenotype in individuals with T2DM.

Four synthetic DNA templates were developed to validate the primers and probes used in the qPCR. These templates were designed as double-stranded DNA (dsDNA) and included segments of the tRNA^leu^ gene and the ATP6 gene containing the target mutation sites. The dsDNA templates representing the normal and 3243 mutation conditions were 511 bp in length, spanning positions 3090 to 3600, with the A at position 3243 substituted with G in the mutant dsDNA. Similarly, the dsDNA templates for the normal and 9053 mutation conditions were 274 bp long, spanning positions 8887 to 9160, with the G at position 9053 replaced by A in the mutant dsDNA.

**Figure 1.**
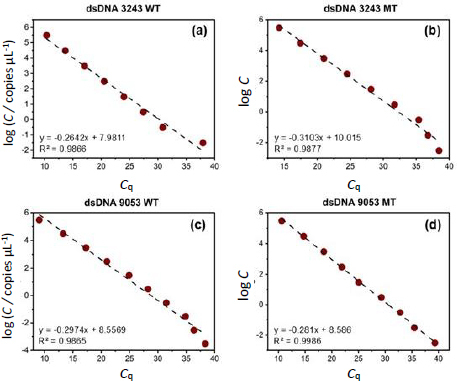
qPCR standard curves for wild-type and mutant dsDNA templates of A3243G and G9053A mutations

The qPCR results for the four dsDNA templates were satisfactory, as evidenced by the varying *C*_q_ values obtained at different concentrations and the strong regression values of the standard curves generated (Figure 1). In these curves, log *C* represents the logarithm of the initial target DNA concentration (copies/μL). These findings confirm that the primers and probes used in the qPCR were highly sensitive and optimal for detecting the target mutations. The mutation levels in each sample were calculated based on the standard curves, revealing that the A3243G mutation levels were generally higher than those of G9053A. However, some samples exhibited higher mutation levels for G9053A. Samples with elevated mutation levels for both A3243G and G9053A were associated with low ATP levels, likely due to increased impairments in ATP synthesis during oxidative phosphorylation.

### Quantification of ATP levels in patient samples using an electrochemical aptasensor

ATP levels were quantified in the T2DM + MD phenotype group (Figure S3) and the T2DM group (Figure S4), showing variations in ATP concentrations between blood and urine samples, demonstrating a strong aptamer response across all samples. In the T2DM + MD phenotype group, the highest peak current in blood samples was 13.247 μA, corresponding to the lowest ATP concentration of 816.9 μM in patient F16. Conversely, the lowest peak current was 2.137 μA, corresponding to the highest ATP concentration of 1712.9 μM in patient F29 (Figure S3). In urine samples, the highest peak current was 19.486 μA, corresponding to the lowest ATP concentration of 313.9 μM in patient F17, while the lowest peak current was 3.920 μA, corresponding to the highest ATP concentration of 1569.2 μM in patient F27 (Table S1).

In the T2DM group, the highest peak current in blood samples was 14.491 μA, corresponding to the lowest ATP concentration of 716.6 μM in sample NF22, while the lowest peak current was 4.351 μA, associated with the highest ATP concentration of 1534.4 μM in sample NF7 (Figure S4). For urine samples, the highest peak current was 15.134 μA, corresponding to the lowest ATP concentration of 664.8 μM in sample NF24, whereas the lowest peak current was 2.030 μA, associated with the highest ATP concentration of 1721.6 μM in sample NF7 (Table S2).

The voltammograms exhibited an inverse correlation between peak current and ATP concentration. As the ATP concentration increased, more ATP molecules bound to the aptamer, resulting in surface crowding on the SPCE and impeding electron transfer between the ferricyanide redox probe and the electrode, which led to a decrease in peak current. ATP concentrations measured in blood and urine samples from T2DM patients were below the normal range of 1.5 to 1.9 mM [28]. Preliminary selectivity tests conducted in a previous study confirmed that the aptasensor effectively differentiated ATP from structurally similar nucleotides (UTP, CTP, and GTP), thereby supporting the specificity of the responses observed in complex biological matrices [25].

### Correlation between the presence of the mutation and ATP levels

Statistical analyses were conducted to examine the relationship between mtDNA mutations at positions 3243 and 9053 and ATP concentrations in blood and urine, with a particular focus on the T2DM and T2DM + MD phenotype groups. Mutations at positions 3243 and 9053 were identified based on the relative Cq values of mutant (FAM) and normal (HEX) DNA, and patients were categorized as either "mutated" or "not mutated" at each site.

The first analysis aimed to determine if ATP levels differed between the T2DM + MD phenotype and T2DM groups. One-way ANOVA was applied, with residual normality and homogeneity of variances validated using the Shapiro-Wilk and Levene’s tests (Table S3 and Table S4). The null hypothesis (H_o_), stating no effect of phenotype on ATP levels, was accepted for both blood and urine samples (*p*-values of 0.366 and 0.261, respectively). These results indicate that differences in ATP concentrations are not attributable solely to the diabetic phenotype.

A two-way ANOVA (Type III model) was performed to evaluate the effects of mutations at positions 3243 and 9053 on ATP concentrations in blood and urine samples. Statistical assumptions of residual normality and homogeneity of variances were met, as confirmed by the Shapiro-Wilk and Levene’s tests (Table 4).

**Table 4.** The Shapiro Wilk test and Levene's test results of blood and urine variance of the T2DM + MD and T2DM groups

Variants	Shapiro-Wilk test		Levene’s test	
	W-test	*p*-value	F-test	*p*-value
T2DM blood + MD phenotype	0.95775	0.271	0.9135	0.448
T2DM urine + MD phenotype	0.92241	0.07503	0.4852	0.6965
Blood T2DM	0.96931	0.5411	0.4475	0.5092
Urine T2DM	0.9715	0.6009	1.3129	0.2863

The analysis revealed a significant association between the presence of mtDNA mutations and ATP levels. Samples harboring mutations at both positions 3243 and 9053 exhibited substantially lower ATP concentrations, consistent with the impaired mitochondrial bioenergetics observed in mitochondrial disorders. This reduction in ATP is attributable to the disruption of oxidative phosphorylation processes, likely caused by structural and functional perturbations in the mitochondrial electron transport chain due to these mutations.

The analysis of variance data for the blood and urine samples from both the T2DM + MD phenotype and T2DM groups are presented in Table 5. Correlation analysis was performed to examine whether ATP concentrations were influenced by the *C*_q_ values, which serve as a mutation marker. The results showed no significant difference in ATP levels between the groups. It is important to note that the lack of significant differences may stem from the reliance on interviews for data collection, which could introduce biases or inaccuracies. Therefore, it is essential to approach the interpretation of these findings with extra caution and consider more precise, validated methods for data collection and analysis. However, the A3243G mutation was found to affect ATP concentrations, particularly in the blood of the T2DM + MD phenotype group and in the urine of the T2DM group. Conversely, the G9053A mutation did not show a similar effect on ATP concentrations in either group.

**Table 5.** Blood and urine variance analysis data of T2DM + MD phenotype and T2DM groups

	Square of variance	Degrees of freedom	Sum of squares	*F*	*p*-value	Conclusion
T2DM blood + DM phenotype	3243	1	96729	2.2159	0.14818	1. H_01_ is accepted (*p* > 0.05) 2. H_02_ is rejected (*p* > 0.05)
	9053	1	228670	5.2385	0.03014	
	Residual	27	2469346			
T2DM urine + DM phenotype	3243	1	30090	0.3733	0.5481	1. H_01_ is accepted (*p* > 0.05) 2. H_02_ is accepted (*p* > 0.05)
	9053	1	9945	0.1234	0.7291	
	Residual	20	1612290			
T2DM blood	3243	1	3833	0.1024	0.7514	H_0_ is accepted (*p* > 0.05)
	Residual	1	1010321			
T2DM urine	3243	1	331239	5.1079	0.03242	1. H_01_ is rejected (*p* > 0.05) 2. H_02_ is accepted (*p* > 0.05)
	9053	1	4852	0.0748	0.78661	
	Residual	26	1686068			

All ATP levels measured from patient samples were below the physiological reference range. However, no statistically significant difference was observed between the T2DM and T2DM + MD phenotype groups. This outcome appears inconsistent with the initial hypothesis that mitochondrial mutations would lead to markedly lower ATP levels in the phenotype group. Despite this, descriptive patterns reveal otherwise. The T2DM + MD phenotype group exhibited a lower ATP concentration range (314 to 919 μM), while the non-phenotype group ranged between 0.746 to 1.130 mM (Table 6). A similar trend was seen in the A3243G mutation levels, which were consistently higher in the phenotype group (61.49 to 87.31 %) compared to the non-phenotype group (53.18 to 63.61 %). In contrast, G9053A mutation levels showed considerable overlap between groups (phenotype: 79.95 to 97.41 %; non-phenotype: 85.17 to 97.86 %), suggesting a weaker contribution to ATP variation.

**Table 6.** Correlation between *C*_q_ values and mutation levels of A3243G and G9053A from T2DM + MD phenotype patient group.

Group	Patient’s code	ATP Level, μM	Mutation level, %	
			A3243G	G9053A
T2DM + MD phenotype patient group	F9	919	87.31	83.49
	F16	681	64.68	83.48
	F17	314	62.50	79.95
	F18	876	61.49	97.41
	F19	887	62.90	85.68
T2DM patient group	NF13	1130	54.21	85.17
	NF15	1041	55.41	93.19
	NF17	746	63.61	87.92
	NF26	1077	54.14	97.86
	NF30	1008	53.18	87.27

These observations were further supported by data in Table S5 and Table S6, in which ATP concentrations tended to remain below 1000 μM in the phenotype group and below 1100 μM in the non-phenotype group. Although these ranges suggest a phenotypic effect, the absence of a statistically significant difference may reflect the influence of several confounding factors. One major consideration is the heteroplasmic nature of mitochondrial mutations, particularly A3243G, which could lead to inconsistent functional impact across individuals depending on mutation load and tissue distribution. Since ATP was measured in blood and urine, which may not directly reflect intracellular mitochondrial function, variability between samples is expected.

Additionally, G9053A remains classified as a secondary mutation, and its heteroplasmic or homoplasmic status was not definitively determined in this study, limiting the interpretability of its impact. Moreover, while qPCR was used to estimate mutation levels, the method may not yet be fully optimized to detect subtle variations in heteroplasmy that could influence ATP synthesis. On the other hand, the aptasensor effectively differentiated ATP levels between diabetic and non-diabetic individuals in preliminary validation, but may not have the resolution to distinguish finer subgroup differences.

Taken together, these findings suggest that the A3243G mutation may play a more pronounced role in modulating ATP levels in mitochondrial diabetes, while the impact of G9053A remains uncertain. This work may serve as a preliminary investigation, and further studies with larger sample sizes, enhanced molecular tools, and tissue-specific analyses are recommended to explore these relationships more definitively.

## Conclusions

The findings of this study reinforce the potential role of mitochondrial DNA mutations in disrupting ATP homeostasis in type 2 diabetes mellitus. A higher prevalence of A3243G and G9053A mutations was observed in participants with mitochondrial disease phenotypes (12/30), compared to those without (6/30). Among these, A3243G showed consistently higher mutation levels, averaging 70.04 % in blood and 63.23% in urine, than G9053A, which averaged 25.84 and 19.10 %, respectively. Electrochemical measurements confirmed ATP concentrations were below the physiological range (1.500 to 1.900 mM) in all patient samples. Descriptively, the T2DM + MD group exhibited lower ATP ranges (314 to 919 μM) than the non-phenotype group (0.746 to 1.130 mM), although the difference was not statistically significant. Two-way ANOVA, however, indicated that the presence of A3243G and G9053A was significantly associated with ATP reduction. The lowest ATP levels were generally found in individuals carrying both mutations, suggesting a potential additive impact on mitochondrial bioenergetics. These results highlight A3243G as a more consistent contributor to ATP deficiency in the context of mitochondrial diabetes, while the role of G9053A remains to be clarified in future studies involving larger, well-stratified cohorts.

## Supplementary material

Additional data are available at https://pub.iapchem.org/ojs/index.php/admet/article/view/2767, or from the corresponding author on request.



## List of abbreviations

ATP: Adenosine triphosphate
BHQ: Black hole quencher
BMI: Body mass index
Cq: Quantification cycle
DM: Diabetes mellitus
DPV: Differential pulse voltammetry
FAM: Fluorescein amidite
HEX: Hexachlorofluorescein
MD: Mitochondrial disease
MELAS: mitochondrial encephalopathy, lactic acidosis, and stroke-like episodes
MID: Maternally inherited diabetes
MIDD: Maternally inherited diabetes and deafness
mtDNA: Mitochondrial DNA
OXPHOS: Oxidative phosphorylation
PCR-RFLP: Polymerase chain reaction-restriction fragment length polymorphism
qPCR: Quantitative polymerase chain reaction
rRNA: Ribosomal RNA
ROS: Reactive oxygen species
SPCE: Screen-printed carbon electrode
tRNA: Transfer RNA
