# Supplementary material for: Correlation between A3243G and G9053A mtDNA mutations and ATP levels in diabetes mellitus patients using qPCR and electrochemical aptasensors
Source: ADMET DMPK. 2025 Jun 12;13(3):2767. doi: 10.5599/admet.2767 (PMC12205922; doi:10.5599/admet.2767)
Supplement: Supplementary file 1 [file ADMET-13-2767-S1.pdf]

ADMET & DMPK 13(3) (2025) S2767

Open Access : ISSN : 1848-7718

http://www.pub.iapchem.org/ojs/index.php/admet/index

Supplementary material to

Correlation between A3243G and G9053A mtDNA mutations and ATP levels in diabetes mellitus patients using qPCR and electrochemical aptasensors

Iman Permana Maksum[1], Rahmaniar Mulyani[1,2], Yeni Wahyuni Hartati[1], Irkham[1], Fanny Rizki Rahmadanthi[1], Serly Zuliska[1] and Toto Subroto[1]

[1]*Department of Chemistry, Faculty of Mathematics and Natural Sciences, Universitas Padjadjaran, Sumedang, 45363, Indonesia*
[2]*Department of Chemistry, Faculty of Sciences and Informatics, Universitas Jendral Achmad Yani, Cimahi, 40525, Indonesia*

ADMET & DMPK **13(3)** (2025) 2667; https://doi.org/10.5599/admet.2767

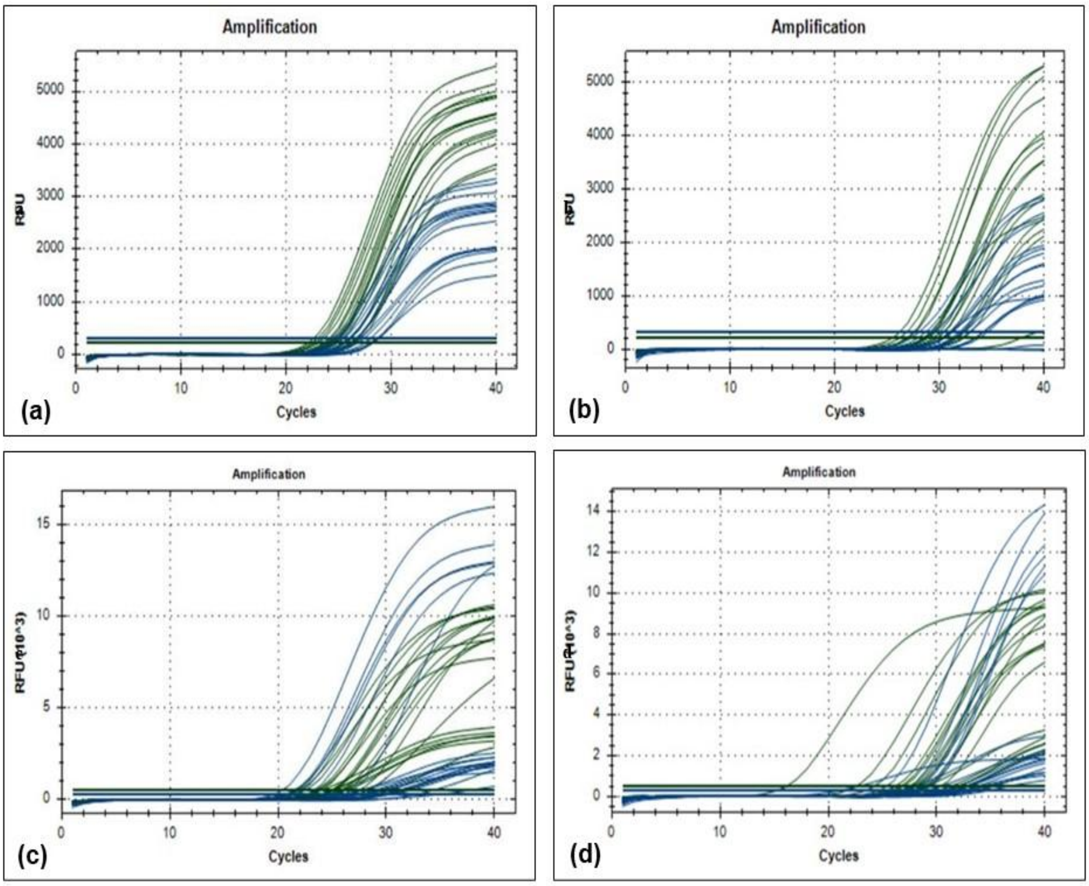

Figure S1. Amplification curves for T2DM + MD phenotype participants (*n* = 30): (a) A3243G mutation detection in blood samples; (b) A3243G mutation detection in urine samples; (c) G9053A mutation detection in blood samples; (d) G9053A mutation detection in urine samples

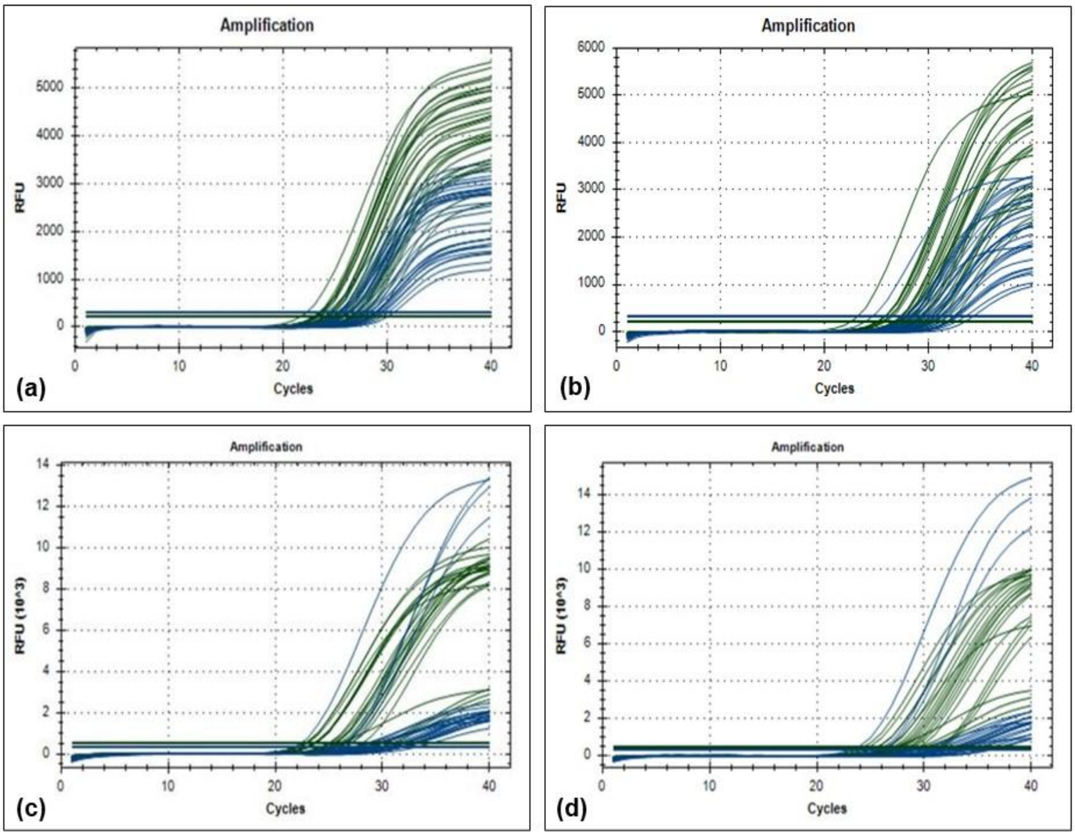

Figure S2. Amplification curves for T2DM participants (*n* = 30): (a) A3243G mutation detection in blood samples; (b) A3243G mutation detection in urine samples; (c) G9053A mutation detection in blood samples; (d) G9053A mutation detection in urine samples

Table S1. Response of the electrochemical aptasensor to 30 blood and urine samples from type 2 diabetes mellitus (T2DM) + MD phenotype patients for ATP detection

No	Patient's code	BLOOD						URINE					
		Height, µA	Area	Width	I_{BARE} / µA*	ΔI(y) / µA	Concentration, µM	Height, µA	Area	Width	I_{BARE} / µA*	ΔI(y) / µA)	Concentration, µM
1	F1	5.43	2.51	0.39	24.26	18.82	1447	3.94	1.42	0.37	24.26	20.31	1567
2	F2	6.01	2.10	0.34	24.26	18.25	1401	5.57	1.79	0.32	24.26	18.68	1436
3	F3	4.97	1.68	0.33	24.26	19.29	1485	6.77	2.86	0.33	24.26	17.49	1340
4	F4	5.61	2.17	0.35	24.26	18.65	1433	5.70	1.92	0.33	24.26	18.56	1426
5	F5	5.79	1.94	0.33	24.26	18.47	1418	5.64	1.79	0.31	24.26	18.61	1430
6	F6	5.03	1.82	0.38	24.26	19.22	1479	8.46	3.51	0.31	24.26	15.79	1203
7	F7	6.38	2.17	0.34	24.26	17.88	1371	6.82	2.29	0.33	24.26	17.43	1335
8	F8	8.99	2.87	0.30	24.26	15.27	1160	11.96	4.46	0.29	24.26	12.30	921
9	F9	11.98	5.19	0.35	24.26	12.28	919	7.38	3.51	0.35	24.26	16.87	1290
10	F10	6.21	2.64	0.37	24.26	18.05	1385	2.26	0.63	0.26	24.26	22.00	1703
11	F11	5.34	2.84	0.37	24.26	18.92	1455	8.50	3.80	0.33	24.26	15.75	1200
12	F12	6.29	2.95	0.36	24.26	17.96	1378	8.90	3.59	0.30	24.26	15.36	1168
13	F13	9.99	4.02	0.29	24.26	14.26	1079	5.38	1.78	0.33	24.26	18.87	1451
14	F14	8.06	2.80	0.31	24.26	16.20	1235	6.04	2.79	0.36	24.26	18.22	1398
15	F15	10.69	3.97	0.31	24.26	13.57	1023	16.48	5.05	0.24	24.26	7.77	556
16	F16	13.25	3.86	0.25	24.26	11.01	817	14.94	4.97	0.25	24.26	9.32	681
17	F17	10.53	3.50	0.29	24.26	13.72	1036	19.49	5.48	0.21	24.26	4.77	314
18	F18	12.51	3.68	0.25	24.26	11.74	876	7.82	3.44	0.33	24.26	16.44	1255
19	F19	12.38	4.60	0.28	24.26	11.88	887	9.68	3.98	0.31	24.26	14.58	1105
20	F20	7.55	3.57	0.35	24.26	16.71	1277	5.73	2.80	0.37	24.26	18.53	1423
21	F21	7.35	3.48	0.35	24.26	16.90	1292	8.82	3.80	0.32	24.26	15.44	1174
22	F22	6.23	3.35	0.38	24.26	18.02	1383	8.07	3.60	0.33	24.26	16.18	1235
23	F23	9.40	3.31	0.31	24.26	14.85	1127	8.46	3.54	0.31	24.26	15.79	1203
24	F24	7.75	3.40	0.33	24.26	16.50	1260	12.10	3.96	0.25	24.26	12.15	909
25	F25	9.69	4.07	0.31	24.26	14.57	1104	9.73	4.00	0.30	24.26	14.53	1101
26	F26	7.89	3.19	0.30	24.26	16.37	1249	8.94	3.72	0.31	24.26	15.31	1164
27	F27	11.52	3.75	0.27	24.26	12.73	956	3.92	1.35	0.36	24.26	20.34	1569
28	F28	6.17	2.43	0.35	24.26	18.09	1388	5.42	2.95	0.40	24.26	18.84	1448
29	F29	2.14	0.31	0.07	24.26	22.12	1713	4.46	1.66	0.37	24.26	19.79	1526
30	F30	5.00	1.83	0.37	24.26	19.26	1482	5.03	1.82	0.38	24.26	19.22	1479

*baseline current responseof the unmodified (bare) electrode measured before aptamer immobilization

(cc) BY

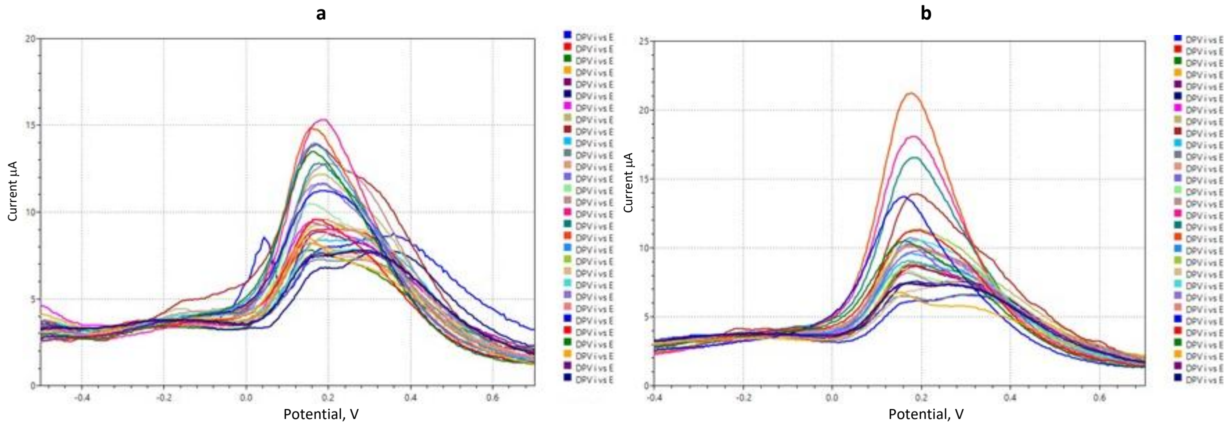

Figure S3. Voltammograms of the aptasensor response of the blood (a) and urine (b) samples of T2DM patients with the MD phenotype

Table S2. Response of the electrochemical aptasensor to 30 blood and urine samples from T2DM patients for ATP detection

No	Patient's code	BLOOD						URINE					
		Height, µA	Area	Width	I_{BARE} / µA*	ΔI(y) / µA	Concentration, µM	Height, µA	Area	Width	I_{BARE} / µA*	ΔI(y) / µA	Concentration, µM
1	NF1	4.65	1.70	0.37	24.26	19.60	1510	5.20	1.77	0.34	24.26	19.06	1466
2	NF2	6.69	3.31	0.36	24.26	17.57	1346	4.48	1.51	0.34	24.26	19.77	1524
3	NF3	5.90	2.10	0.35	24.26	18.35	1409	5.75	1.92	0.33	24.26	18.51	1422
4	NF4	8.25	3.05	0.32	24.26	16.00	1220	4.56	1.72	0.37	24.26	19.70	1518
5	NF5	5.83	2.07	0.35	24.26	18.42	1415	4.02	1.51	0.37	24.26	20.24	1561
6	NF6	6.96	2.35	0.33	24.26	17.30	1324	6.15	2.00	0.33	24.26	18.11	1390
7	NF7	4.35	1.71	0.38	24.26	19.90	1534	2.03	0.61	0.38	24.26	22.23	1722
8	NF8	8.35	3.93	0.33	24.26	15.91	1212	8.03	3.16	0.30	24.26	16.22	1237
9	NF9	6.80	2.27	0.34	24.26	17.46	1337	9.79	3.72	0.29	24.26	14.46	1095
10	NF10	7.93	2.98	0.33	24.26	16.33	1246	11.73	4.33	0.27	24.26	12.52	939
11	NF11	11.28	3.42	0.27	24.26	12.97	976	10.68	4.26	0.30	24.26	13.58	1024
13	NF13	8.47	3.94	0.33	24.26	15.79	1202	9.36	3.84	0.31	24.26	14.90	1130
14	NF14	10.51	4.30	0.29	24.26	13.75	1038	10.44	3.97	0.28	24.26	13.81	1043
15	NF15	10.47	3.77	0.27	24.26	13.78	1041	7.78	3.52	0.35	24.26	16.48	1258
16	NF16	8.04	3.68	0.34	24.26	16.21	1237	10.12	3.97	0.30	24.26	14.13	1069
17	NF17	6.66	2.80	0.36	24.26	17.60	1349	14.13	4.71	0.26	24.26	10.13	746
18	NF18	11.20	4.26	0.29	24.26	13.05	982	13.06	4.48	0.26	24.26	11.20	832
19	NF19	11.83	4.42	0.29	24.26	12.43	931	11.99	4.48	0.28	24.26	12.26	918
20	NF20	10.23	4.09	0.31	24.26	14.02	1060	11.25	3.96	0.27	24.26	13.00	978
21	NF21	8.79	2.83	0.30	24.26	15.47	1177	7.54	2.30	0.29	24.26	16.72	1277
22	NF22	14.49	4.15	0.24	24.26	9.76	717	9.25	3.04	0.27	24.26	15.00	1139
23	NF23	7.93	2.56	0.31	24.26	16.32	1246	10.72	3.93	0.29	24.26	13.53	1021
24	NF24	8.60	2.76	0.30	24.26	15.65	1191	15.13	4.75	0.24	24.26	9.12	665
25	NF25	6.56	2.54	0.34	24.26	17.69	1356	12.38	4.61	0.28	24.26	11.87	887
26	NF26	7.50	3.62	0.35	24.26	16.76	1280	10.03	4.09	0.29	24.26	14.23	1077
27	NF27	9.18	3.24	0.30	24.26	15.08	1145	12.66	4.62	0.27	24.26	11.59	864
28	NF28	11.39	4.62	0.29	24.26	12.86	967	12.91	4.69	0.27	24.26	11.35	845
29	NF29	6.29	2.29	0.33	24.26	17.97	1378	6.51	3.14	0.36	24.26	17.74	1360
30	NF30	10.88	4.50	0.30	24.26	13.37	1008	7.44	3.20	0.33	24.26	16.82	1286

*baseline current responseof the unmodified (bare) electrode measured before aptamer immobilization

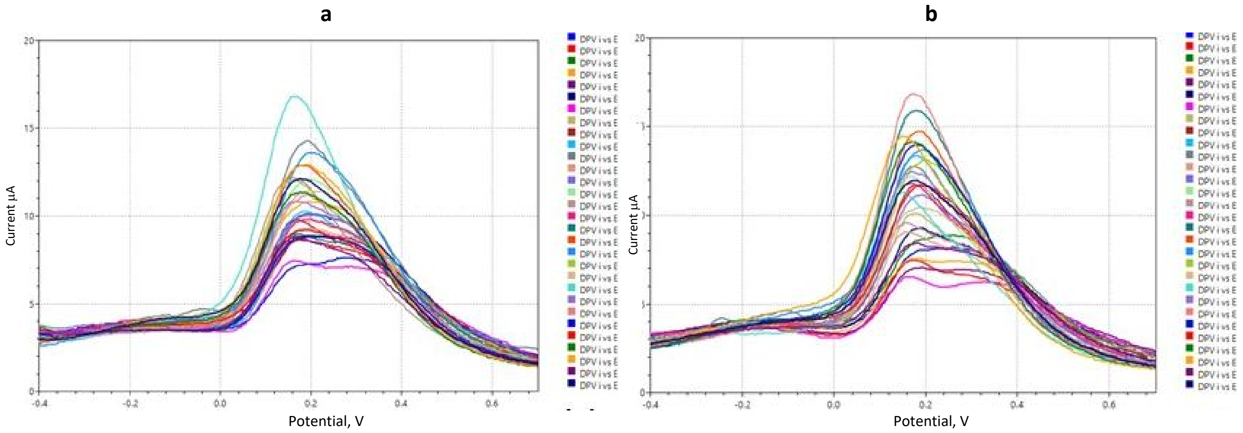

Figure S4. Voltammogram of electrochemical aptasensor response of the blood (a) and urine (b) samples of T2DM patients

Table S3. Blood variance analysis

Source of variation	Degrees of freedom	Sum of squares	Mean square	*F*-value	*p*-value
Phenotype	1	36041	36041	0.832	0.366
Residual	57	246924	4332		

Table S4. Urine variance analysis

Source of variation	Degrees of freedom	Sum of squares	Mean square	*F*-value	*p*-value
Phenotype	1	108078	108078	1.288	0.261
Residual	57	4783397	83919		

Table S5. Correlation between Cq values and mutation levels of A3243G and G9053A from T2DM + MD phenotype patient group.

Number	Patient's code	ATP level, μM	Mutation level, %	
			A3243G	G9053A
1	F1	1447	58.58	1.76
2	F2	1401	73.76	1.37
3	F3	1340	59.40	8.45
4	F4	1426	71.02	18.02
5	F5	1418	53.16	1.64
6	F6	1203	53.16	54.47
7	F7	1335	66.55	1.58
8	F8	921	66.76	1.99
9	F9	919	87.31	83.49
10	F10	1385	76.82	17.15
11	F11	1200	60.95	8.44
12	F12	1168	65.29	1.89
13	F13	1079	55.01	8.60
14	F14	1235	56.99	7.00
15	F15	556	65.93	1.50
16	F16	681	64.68	83.48
17	F17	314	62.50	79.95
18	F18	876	61.49	97.41
19	F19	887	62.90	85.68
20	F20	1277	55.99	1.71
21	F21	1174	76.62	1.45
22	F22	1235	95.92	1.62
23	F23	1127	88.98	1.47
24	F24	909	53.47	1.63
25	F25	1101	63.05	2.03
26	F26	1164	92.06	84.40
27	F27	956	52.53	0.29
28	F28	1388	89.25	2.42
29	F29	1526	98.65	83.11
30	F30	1479	91.64	1.20

Table S6. Correlation between *Cq* values and mutation levels of A3243G and G9053A from T2DM patient group

Number	Patient's code	ATP Level, μM	Mutation level, %	
			A3243G	A9053G
1	NF1	1466	57.33	0.13
2	NF2	1436	60.69	1.75
3	NF3	1422	60.60	1.61
4	NF4	1220	62.39	1.83
5	NF5	1415	55.81	1.18
6	NF6	1324	54.21	0.93
7	NF7	1534	98.20	1.27
8	NF8	1212	74.62	1.07
9	NF9	1095	71.02	18.02
10	NF10	939	66.99	8.65
11	NF11	976	55.73	1.32
12	NF13	1130	54.21	85.17
13	NF14	1038	65.54	1.23

CC BY

Number	Patient's code	ATP Level, µM	Mutation level, %	
			A3243G	A9053G
14	NF15	1041	55.41	93.19
15	NF16	1069	64.21	1.65
16	NF17	746	63.61	87.92
17	NF18	832	65.44	1.88
18	NF19	918	52.18	9.32
19	NF20	978	67.70	1.11
20	NF21	1177	60.98	7.92
21	NF22	717	63.79	1.59
22	NF23	1021	58.55	8.98
23	NF24	665	64.65	7.47
24	NF25	887	63.40	2.18
25	NF26	1077	54.14	97.86
26	NF27	864	62.44	7.69
27	NF28	845	54.64	11.91
28	NF29	1360	71.92	1.75
29	NF30	1008	53.18	87.27
