# Supplementary material for: Correlation between A3243G and G9053A mtDNA mutations and ATP levels in diabetes mellitus patients using qPCR and electrochemical aptasensors
Source: ADMET DMPK. 2025 Jun 12;13(3):2767. doi: 10.5599/admet.2767 (PMC12205922; doi:10.5599/admet.2767)
Supplement: Supplementary file 2 [file ADMET-13-2767-S1.docx]

*ADMET & DMPK 13(3) (2025) S2767*

*
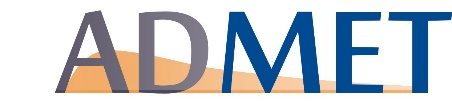
***Open Access : ISSN : 1848-7718**[***http://www.pub.iapchem.org/ojs/index.php/admet/index***](http://www.pub.iapchem.org/ojs/index.php/admet/index)

Supplementary material to

**Correlation between A3243G and G9053A mtDNA mutations and ATP levels in diabetes mellitus patients using qPCR and electrochemical aptasensors**

Iman Permana Maksum^1^, Rahmaniar Mulyani^1,2^, Yeni Wahyuni Hartati^1^, Irkham^1^, Fanny Rizki Rahmadanthi^1^, Serly Zuliska^1^ and Toto Subroto^1^

^1^Department of Chemistry, Faculty of Mathematics and Natural Sciences, Universitas Padjadjaran, Sumedang, 45363, Indonesia
^2^Department of Chemistry, Faculty of Sciences and Informatics, Universitas Jendral Achmad Yani, Cimahi, 40525, Indonesia

ADMET & DMPK **13(3)** (2025) 2667; <https://doi.org/10.5599/admet.2767>


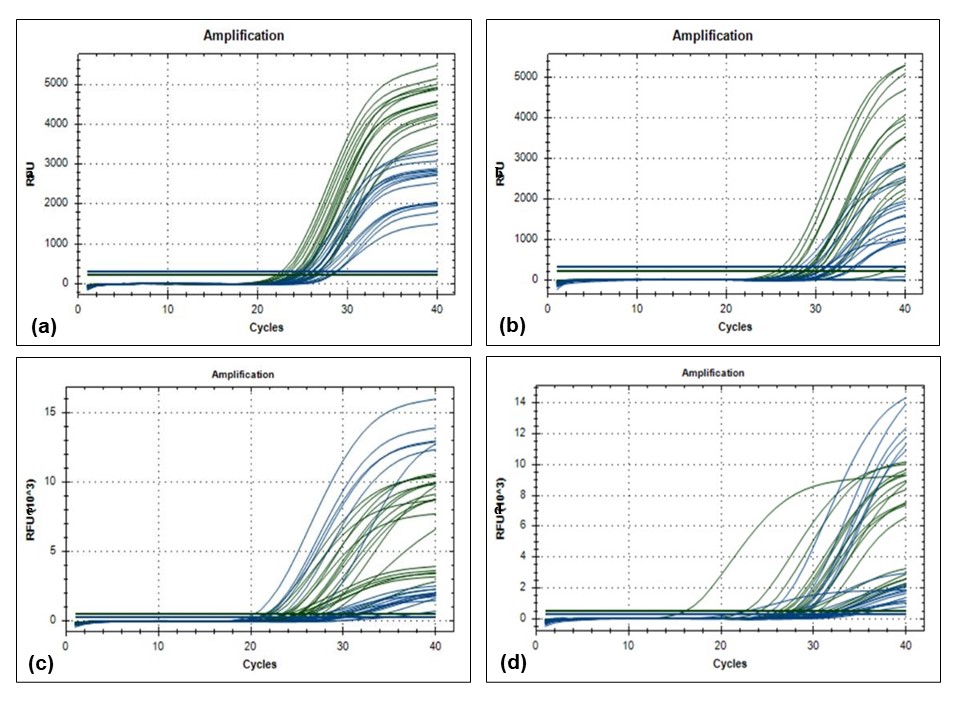


**Figure S1.** Amplification curves for T2DM + MD phenotype participants (*n* = 30): (a) A3243G mutation detection in blood samples; (b) A3243G mutation detection in urine samples; (c) G9053A mutation detection in blood samples; (d) G9053A mutation detection in urine samples


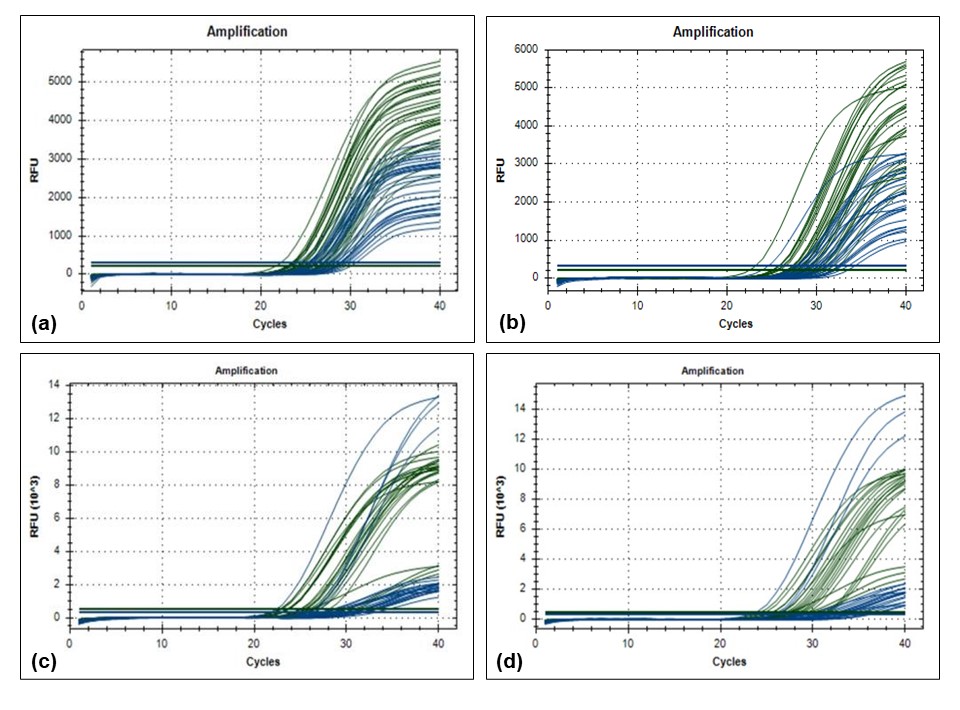


**Figure S2.** Amplification curves for T2DM participants (*n* = 30): (a) A3243G mutation detection in blood samples; (b) A3243G mutation detection in urine samples; (c) G9053A mutation detection in blood samples; (d) G9053A mutation detection in urine samples

**Table S1.** Response of the electrochemical aptasensor to 30 blood and urine samples from type 2 diabetes mellitus (T2DM) + MD phenotype patients for ATP detection

| No | Patient’s code | BLOOD | | | | | | URINE | | | | | |
| --- | --- | --- | --- | --- | --- | --- | --- | --- | --- | --- | --- | --- | --- |
|  |  | Height, µA | Area | Width | *I*_BARE_ / μA* | Δ*I*(y) / µA | Concentration, µM | Height, µA | Area | Width | *I*_BARE_ / μA* | Δ*I*(y) / µA) | Concentration, µM |
| 1 | F1 | 5.43 | 2.51 | 0.39 | 24.26 | 18.82 | 1447 | 3.94 | 1.42 | 0.37 | 24.26 | 20.31 | 1567 |
| 2 | F2 | 6.01 | 2.10 | 0.34 | 24.26 | 18.25 | 1401 | 5.57 | 1.79 | 0.32 | 24.26 | 18.68 | 1436 |
| 3 | F3 | 4.97 | 1.68 | 0.33 | 24.26 | 19.29 | 1485 | 6.77 | 2.86 | 0.33 | 24.26 | 17.49 | 1340 |
| 4 | F4 | 5.61 | 2.17 | 0.35 | 24.26 | 18.65 | 1433 | 5.70 | 1.92 | 0.33 | 24.26 | 18.56 | 1426 |
| 5 | F5 | 5.79 | 1.94 | 0.33 | 24.26 | 18.47 | 1418 | 5.64 | 1.79 | 0.31 | 24.26 | 18.61 | 1430 |
| 6 | F6 | 5.03 | 1.82 | 0.38 | 24.26 | 19.22 | 1479 | 8.46 | 3.51 | 0.31 | 24.26 | 15.79 | 1203 |
| 7 | F7 | 6.38 | 2.17 | 0.34 | 24.26 | 17.88 | 1371 | 6.82 | 2.29 | 0.33 | 24.26 | 17.43 | 1335 |
| 8 | F8 | 8.99 | 2.87 | 0.30 | 24.26 | 15.27 | 1160 | 11.96 | 4.46 | 0.29 | 24.26 | 12.30 | 921 |
| 9 | F9 | 11.98 | 5.19 | 0.35 | 24.26 | 12.28 | 919 | 7.38 | 3.51 | 0.35 | 24.26 | 16.87 | 1290 |
| 10 | F10 | 6.21 | 2.64 | 0.37 | 24.26 | 18.05 | 1385 | 2.26 | 0.63 | 0.26 | 24.26 | 22.00 | 1703 |
| 11 | F11 | 5.34 | 2.84 | 0.37 | 24.26 | 18.92 | 1455 | 8.50 | 3.80 | 0.33 | 24.26 | 15.75 | 1200 |
| 12 | F12 | 6.29 | 2.95 | 0.36 | 24.26 | 17.96 | 1378 | 8.90 | 3.59 | 0.30 | 24.26 | 15.36 | 1168 |
| 13 | F13 | 9.99 | 4.02 | 0.29 | 24.26 | 14.26 | 1079 | 5.38 | 1.78 | 0.33 | 24.26 | 18.87 | 1451 |
| 14 | F14 | 8.06 | 2.80 | 0.31 | 24.26 | 16.20 | 1235 | 6.04 | 2.79 | 0.36 | 24.26 | 18.22 | 1398 |
| 15 | F15 | 10.69 | 3.97 | 0.31 | 24.26 | 13.57 | 1023 | 16.48 | 5.05 | 0.24 | 24.26 | 7.77 | 556 |
| 16 | F16 | 13.25 | 3.86 | 0.25 | 24.26 | 11.01 | 817 | 14.94 | 4.97 | 0.25 | 24.26 | 9.32 | 681 |
| 17 | F17 | 10.53 | 3.50 | 0.29 | 24.26 | 13.72 | 1036 | 19.49 | 5.48 | 0.21 | 24.26 | 4.77 | 314 |
| 18 | F18 | 12.51 | 3.68 | 0.25 | 24.26 | 11.74 | 876 | 7.82 | 3.44 | 0.33 | 24.26 | 16.44 | 1255 |
| 19 | F19 | 12.38 | 4.60 | 0.28 | 24.26 | 11.88 | 887 | 9.68 | 3.98 | 0.31 | 24.26 | 14.58 | 1105 |
| 20 | F20 | 7.55 | 3.57 | 0.35 | 24.26 | 16.71 | 1277 | 5.73 | 2.80 | 0.37 | 24.26 | 18.53 | 1423 |
| 21 | F21 | 7.35 | 3.48 | 0.35 | 24.26 | 16.90 | 1292 | 8.82 | 3.80 | 0.32 | 24.26 | 15.44 | 1174 |
| 22 | F22 | 6.23 | 3.35 | 0.38 | 24.26 | 18.02 | 1383 | 8.07 | 3.60 | 0.33 | 24.26 | 16.18 | 1235 |
| 23 | F23 | 9.40 | 3.31 | 0.31 | 24.26 | 14.85 | 1127 | 8.46 | 3.54 | 0.31 | 24.26 | 15.79 | 1203 |
| 24 | F24 | 7.75 | 3.40 | 0.33 | 24.26 | 16.50 | 1260 | 12.10 | 3.96 | 0.25 | 24.26 | 12.15 | 909 |
| 25 | F25 | 9.69 | 4.07 | 0.31 | 24.26 | 14.57 | 1104 | 9.73 | 4.00 | 0.30 | 24.26 | 14.53 | 1101 |
| 26 | F26 | 7.89 | 3.19 | 0.30 | 24.26 | 16.37 | 1249 | 8.94 | 3.72 | 0.31 | 24.26 | 15.31 | 1164 |
| 27 | F27 | 11.52 | 3.75 | 0.27 | 24.26 | 12.73 | 956 | 3.92 | 1.35 | 0.36 | 24.26 | 20.34 | 1569 |
| 28 | F28 | 6.17 | 2.43 | 0.35 | 24.26 | 18.09 | 1388 | 5.42 | 2.95 | 0.40 | 24.26 | 18.84 | 1448 |
| 29 | F29 | 2.14 | 0.31 | 0.07 | 24.26 | 22.12 | 1713 | 4.46 | 1.66 | 0.37 | 24.26 | 19.79 | 1526 |
| 30 | F30 | 5.00 | 1.83 | 0.37 | 24.26 | 19.26 | 1482 | 5.03 | 1.82 | 0.38 | 24.26 | 19.22 | 1479 |

*baseline current responseof the unmodified (bare) electrode measured before aptamer immobilization

**a b**

Current μA

Current μA


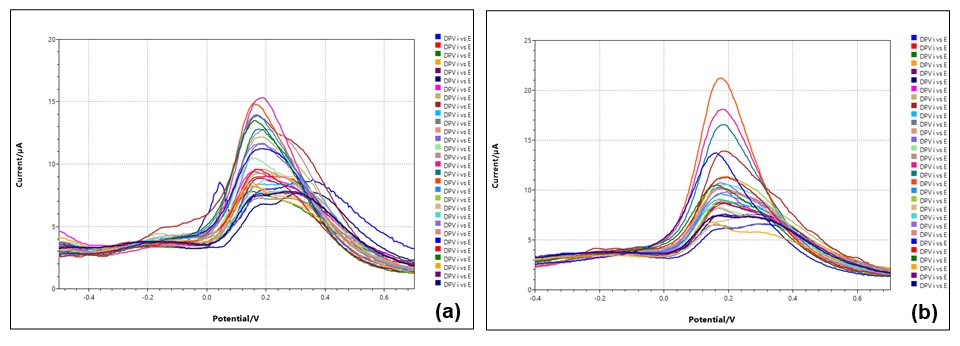

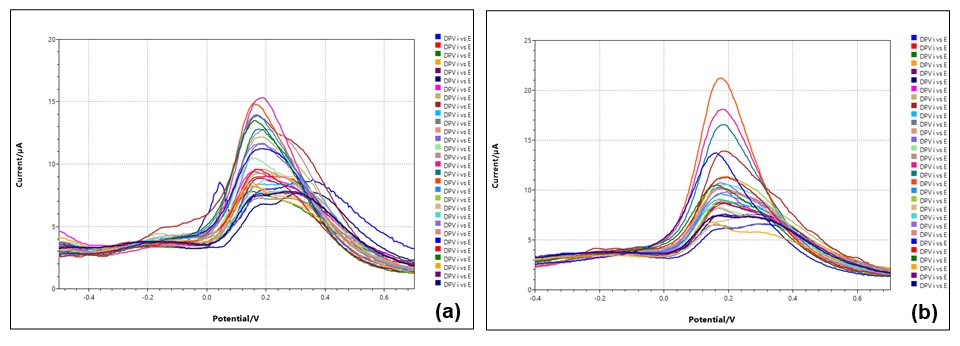


Potential, V Potential, V

**Figure S3.** Voltammograms of the aptasensor response of the blood (a) and urine (b) samples of T2DM patients with the MD phenotype

**Table S2.** Response of the electrochemical aptasensor to 30 blood and urine samples from T2DM patients for ATP detection

| No | Patient’s code | BLOOD | | | | | | URINE | | | | | |
| --- | --- | --- | --- | --- | --- | --- | --- | --- | --- | --- | --- | --- | --- |
|  |  | Height, µA | Area | Width | *I*_BARE_ / μA* | Δ*I*(y) / µA | Concentration, µM | Height, µA | Area | Width | *I*_BARE_ / μA* | Δ*I*(y) / µA | Concentration, µM |
| 1 | NF1 | 4.65 | 1.70 | 0.37 | 24.26 | 19.60 | 1510 | 5.20 | 1.77 | 0.34 | 24.26 | 19.06 | 1466 |
| 2 | NF2 | 6.69 | 3.31 | 0.36 | 24.26 | 17.57 | 1346 | 4.48 | 1.51 | 0.34 | 24.26 | 19.77 | 1524 |
| 3 | NF3 | 5.90 | 2.10 | 0.35 | 24.26 | 18.35 | 1409 | 5.75 | 1.92 | 0.33 | 24.26 | 18.51 | 1422 |
| 4 | NF4 | 8.25 | 3.05 | 0.32 | 24.26 | 16.00 | 1220 | 4.56 | 1.72 | 0.37 | 24.26 | 19.70 | 1518 |
| 5 | NF5 | 5.83 | 2.07 | 0.35 | 24.26 | 18.42 | 1415 | 4.02 | 1.51 | 0.37 | 24.26 | 20.24 | 1561 |
| 6 | NF6 | 6.96 | 2.35 | 0.33 | 24.26 | 17.30 | 1324 | 6.15 | 2.00 | 0.33 | 24.26 | 18.11 | 1390 |
| 7 | NF7 | 4.35 | 1.71 | 0.38 | 24.26 | 19.90 | 1534 | 2.03 | 0.61 | 0.38 | 24.26 | 22.23 | 1722 |
| 8 | NF8 | 8.35 | 3.93 | 0.33 | 24.26 | 15.91 | 1212 | 8.03 | 3.16 | 0.30 | 24.26 | 16.22 | 1237 |
| 9 | NF9 | 6.80 | 2.27 | 0.34 | 24.26 | 17.46 | 1337 | 9.79 | 3.72 | 0.29 | 24.26 | 14.46 | 1095 |
| 10 | NF10 | 7.93 | 2.98 | 0.33 | 24.26 | 16.33 | 1246 | 11.73 | 4.33 | 0.27 | 24.26 | 12.52 | 939 |
| 11 | NF11 | 11.28 | 3.42 | 0.27 | 24.26 | 12.97 | 976 | 10.68 | 4.26 | 0.30 | 24.26 | 13.58 | 1024 |
| 13 | NF13 | 8.47 | 3.94 | 0.33 | 24.26 | 15.79 | 1202 | 9.36 | 3.84 | 0.31 | 24.26 | 14.90 | 1130 |
| 14 | NF14 | 10.51 | 4.30 | 0.29 | 24.26 | 13.75 | 1038 | 10.44 | 3.97 | 0.28 | 24.26 | 13.81 | 1043 |
| 15 | NF15 | 10.47 | 3.77 | 0.27 | 24.26 | 13.78 | 1041 | 7.78 | 3.52 | 0.35 | 24.26 | 16.48 | 1258 |
| 16 | NF16 | 8.04 | 3.68 | 0.34 | 24.26 | 16.21 | 1237 | 10.12 | 3.97 | 0.30 | 24.26 | 14.13 | 1069 |
| 17 | NF17 | 6.66 | 2.80 | 0.36 | 24.26 | 17.60 | 1349 | 14.13 | 4.71 | 0.26 | 24.26 | 10.13 | 746 |
| 18 | NF18 | 11.20 | 4.26 | 0.29 | 24.26 | 13.05 | 982 | 13.06 | 4.48 | 0.26 | 24.26 | 11.20 | 832 |
| 19 | NF19 | 11.83 | 4.42 | 0.29 | 24.26 | 12.43 | 931 | 11.99 | 4.48 | 0.28 | 24.26 | 12.26 | 918 |
| 20 | NF20 | 10.23 | 4.09 | 0.31 | 24.26 | 14.02 | 1060 | 11.25 | 3.96 | 0.27 | 24.26 | 13.00 | 978 |
| 21 | NF21 | 8.79 | 2.83 | 0.30 | 24.26 | 15.47 | 1177 | 7.54 | 2.30 | 0.29 | 24.26 | 16.72 | 1277 |
| 22 | NF22 | 14.49 | 4.15 | 0.24 | 24.26 | 9.76 | 717 | 9.25 | 3.04 | 0.27 | 24.26 | 15.00 | 1139 |
| 23 | NF23 | 7.93 | 2.56 | 0.31 | 24.26 | 16.32 | 1246 | 10.72 | 3.93 | 0.29 | 24.26 | 13.53 | 1021 |
| 24 | NF24 | 8.60 | 2.76 | 0.30 | 24.26 | 15.65 | 1191 | 15.13 | 4.75 | 0.24 | 24.26 | 9.12 | 665 |
| 25 | NF25 | 6.56 | 2.54 | 0.34 | 24.26 | 17.69 | 1356 | 12.38 | 4.61 | 0.28 | 24.26 | 11.87 | 887 |
| 26 | NF26 | 7.50 | 3.62 | 0.35 | 24.26 | 16.76 | 1280 | 10.03 | 4.09 | 0.29 | 24.26 | 14.23 | 1077 |
| 27 | NF27 | 9.18 | 3.24 | 0.30 | 24.26 | 15.08 | 1145 | 12.66 | 4.62 | 0.27 | 24.26 | 11.59 | 864 |
| 28 | NF28 | 11.39 | 4.62 | 0.29 | 24.26 | 12.86 | 967 | 12.91 | 4.69 | 0.27 | 24.26 | 11.35 | 845 |
| 29 | NF29 | 6.29 | 2.29 | 0.33 | 24.26 | 17.97 | 1378 | 6.51 | 3.14 | 0.36 | 24.26 | 17.74 | 1360 |
| 30 | NF30 | 10.88 | 4.50 | 0.30 | 24.26 | 13.37 | 1008 | 7.44 | 3.20 | 0.33 | 24.26 | 16.82 | 1286 |

*baseline current responseof the unmodified (bare) electrode measured before aptamer immobilization

**a b**


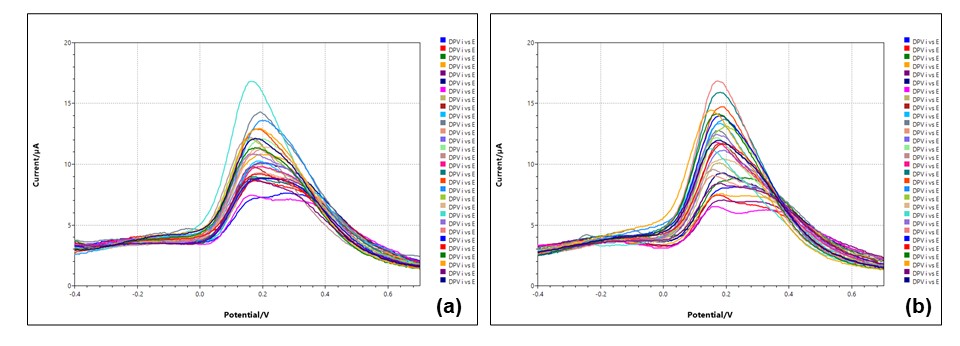

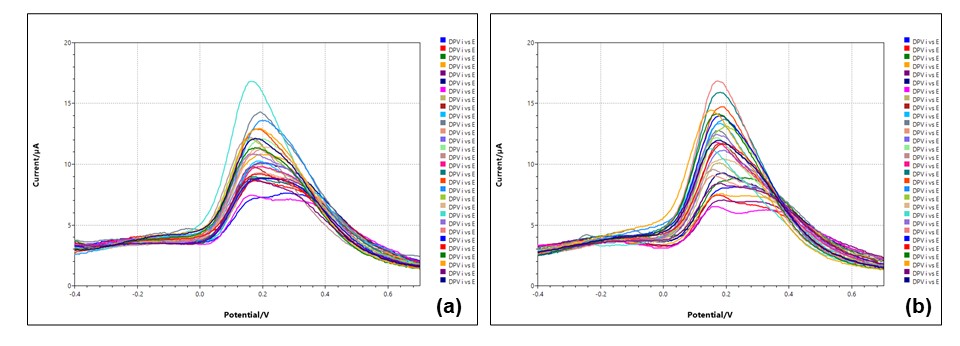


Current μA

Current μA

Potential, V Potential, V

**Figure S4.** Voltammogram of electrochemical aptasensor response of the blood (a) and urine (b) samples of T2DM patients

**Table S3.** Blood variance analysis

| Source of variation | Degrees of freedom | Sum of squares | Mean square | *F*-value | *p*-value |
| --- | --- | --- | --- | --- | --- |
| Phenotype | 1 | 36041 | 36041 | 0.832 | 0.366 |
| Residual | 57 | 246924 | 4332 |  |  |

**Table S4.** Urine variance analysis

| Source of variation | Degrees of freedom | Sum of squares | Mean square | *F*-value | *p*-value |
| --- | --- | --- | --- | --- | --- |
| Phenotype | 1 | 108078 | 108078 | 1.288 | 0.261 |
| Residual | 57 | 4783397 | 83919 |  |  |

**Table S5.** Correlation between Cq values and mutation levels of A3243G and G9053A from T2DM + MD phenotype patient group.

| Number | Patient’s code | ATP level, µM | Mutation level, % | |
| --- | --- | --- | --- | --- |
|  |  |  | A3243G | G9053A |
| 1 | F1 | 1447 | 58.58 | 1.76 |
| 2 | F2 | 1401 | 73.76 | 1.37 |
| 3 | F3 | 1340 | 59.40 | 8.45 |
| 4 | F4 | 1426 | 71.02 | 18.02 |
| 5 | F5 | 1418 | 53.16 | 1.64 |
| 6 | F6 | 1203 | 53.16 | 54.47 |
| 7 | F7 | 1335 | 66.55 | 1.58 |
| 8 | F8 | 921 | 66.76 | 1.99 |
| 9 | F9 | 919 | 87.31 | 83.49 |
| 10 | F10 | 1385 | 76.82 | 17.15 |
| 11 | F11 | 1200 | 60.95 | 8.44 |
| 12 | F12 | 1168 | 65.29 | 1.89 |
| 13 | F13 | 1079 | 55.01 | 8.60 |
| 14 | F14 | 1235 | 56.99 | 7.00 |
| 15 | F15 | 556 | 65.93 | 1.50 |
| 16 | F16 | 681 | 64.68 | 83.48 |
| 17 | F17 | 314 | 62.50 | 79.95 |
| 18 | F18 | 876 | 61.49 | 97.41 |
| 19 | F19 | 887 | 62.90 | 85.68 |
| 20 | F20 | 1277 | 55.99 | 1.71 |
| 21 | F21 | 1174 | 76.62 | 1.45 |
| 22 | F22 | 1235 | 95.92 | 1.62 |
| 23 | F23 | 1127 | 88.98 | 1.47 |
| 24 | F24 | 909 | 53.47 | 1.63 |
| 25 | F25 | 1101 | 63.05 | 2.03 |
| 26 | F26 | 1164 | 92.06 | 84.40 |
| 27 | F27 | 956 | 52.53 | 0.29 |
| 28 | F28 | 1388 | 89.25 | 2.42 |
| 29 | F29 | 1526 | 98.65 | 83.11 |
| 30 | F30 | 1479 | 91.64 | 1.20 |

**Table S6.** Correlation between *Cq* values and mutation levels of A3243G and G9053A from T2DM patient group

| Number | Patient’s code | ATP Level, µM | Mutation level, % | |
| --- | --- | --- | --- | --- |
|  |  |  | A3243G | A9053G |
| 1 | NF1 | 1466 | 57.33 | 0.13 |
| 2 | NF2 | 1436 | 60.69 | 1.75 |
| 3 | NF3 | 1422 | 60.60 | 1.61 |
| 4 | NF4 | 1220 | 62.39 | 1.83 |
| 5 | NF5 | 1415 | 55.81 | 1.18 |
| 6 | NF6 | 1324 | 54.21 | 0.93 |
| 7 | NF7 | 1534 | 98.20 | 1.27 |
| 8 | NF8 | 1212 | 74.62 | 1.07 |
| 9 | NF9 | 1095 | 71.02 | 18.02 |
| 10 | NF10 | 939 | 66.99 | 8.65 |
| 11 | NF11 | 976 | 55.73 | 1.32 |
| 12 | NF13 | 1130 | 54.21 | 85.17 |
| 13 | NF14 | 1038 | 65.54 | 1.23 |
| 14 | NF15 | 1041 | 55.41 | 93.19 |
| 15 | NF16 | 1069 | 64.21 | 1.65 |
| 16 | NF17 | 746 | 63.61 | 87.92 |
| 17 | NF18 | 832 | 65.44 | 1.88 |
| 18 | NF19 | 918 | 52.18 | 9.32 |
| 19 | NF20 | 978 | 67.70 | 1.11 |
| 20 | NF21 | 1177 | 60.98 | 7.92 |
| 21 | NF22 | 717 | 63.79 | 1.59 |
| 22 | NF23 | 1021 | 58.55 | 8.98 |
| 23 | NF24 | 665 | 64.65 | 7.47 |
| 24 | NF25 | 887 | 63.40 | 2.18 |
| 25 | NF26 | 1077 | 54.14 | 97.86 |
| 26 | NF27 | 864 | 62.44 | 7.69 |
| 27 | NF28 | 845 | 54.64 | 11.91 |
| 28 | NF29 | 1360 | 71.92 | 1.75 |
| 29 | NF30 | 1008 | 53.18 | 87.27 |
